# Elevated expression of B7 homolog 4 is associated with disease progression in upper urinary tract urothelial carcinoma

**DOI:** 10.1007/s00262-021-03011-5

**Published:** 2021-07-18

**Authors:** Tomoya Mizuno, Takao Kamai, Toyonori Tsuzuki, Daisaku Nishihara, Toshiki Kijima, Kyoko Arai, Ken-Ichiro Yoshida

**Affiliations:** 1 Department of Urology, Dokkyo Medical University, 880 Kitakobayashi Mibu, Mibu, Tochigi 321-0293 Japan; 2 Department of Surgical Pathology, Aichi Medical University, Nagakute, Aichi Japan

**Keywords:** B7 homolog 4 (B7-H4), CD8, T cell intracellular antigen 1 (TIA-1), Programmed cell death 1 ligand 1 (PD-L1), Tumor-infiltrating lymphocyte (TIL), Upper tract urothelial carcinoma (UTUC)

## Abstract

**Background:**

B7 homolog 4 (B7-H4) is a negative regulator of immune responses, but its immunoregulatory role in the tumor microenvironment of upper urinary tract urothelial carcinoma (UTUC) remains unclear.

**Methods:**

We measured the immunohistochemical expression of B7-H4, CD8 and T cell intracellular antigen 1 (TIA-1), a marker of activated CD8, in 133 patients with UTUC who underwent nephroureterectomy. We also studied the relationship between B7-H4, CD8 and TIA-1 expression and clinicopathological characteristics.

**Results:**

B7-H4 was mainly expressed on the surface in tumor cells, while CD8 and TIA-1 were often expressed in tumor-infiltrating lymphocytes. Elevated expression of B7-H4 in tumor cells was associated with a poorer histological grade, higher pT stage, regional lymph node metastasis, lymphovascular invasion, poorer response of recurrent metastatic lesions to systemic chemotherapy and shorter overall survival. Expression of CD-8 or TIA-1 alone did not correlate directly with clinicopathological characteristics, but among the patients with higher B7-H4 expression in the primary tumors, those with higher CD8 or TIA-1 expression had a better response to systemic chemotherapy, and longer survival, than these with lower CD8 or TIA-1 expression. Cox multivariate regression analysis revealed that higher expression of B7-H4 was associated with shorter overall survival.

**Conclusions:**

These findings suggest that B7-H4 expression in the tumor microenvironment influences the progression of UTUC through cancer immunity and metabolic activity. Tumor cell-associated B7-H4 might be a potential target for cancer immunotherapies.

**Supplementary Information:**

The online version contains supplementary material available at 10.1007/s00262-021-03011-5.

## Introduction

Until recently, less than 10% of all urothelial cancers were upper tract urothelial carcinomas (UTUCs), but this type of cancer is now becoming more common [1]. Treatment for UTUC includes radical nephroureterectomy and chemotherapy. However, the prognosis is poor because of frequent recurrence and metastases; furthermore, treatment resistance to chemotherapy is often seen in metastases, including those in lymph nodes [1–3]. Platinum-based chemotherapy may represent the best treatment option, but its use is controversial because it can damage the kidneys and kidney function is already decreased in patients after radical nephroureterectomy [4, 5].

The immune system is accepted to have dual host-protective and tumor-promoting actions, and cancer cells are known to engage in aerobic glycolysis to meet the intrinsic biosynthetic requirements of cell growth and proliferation. Thus, immune evasion and reprogramming of energy metabolism for the growth and development of tumors are viewed as new hallmarks of cancer [6]. Cancer-induced immunosuppression allows tumors to evade immune surveillance [7–10]. In particular, immune checkpoints play a pivotal role in T cell activation and determine the functional outcome of T cell receptor signaling. The B7 and CD28 families may be key players in the immune checkpoint system by activating and inhibiting co-stimulatory molecules that positively or negatively regulate immune responses [7–10]. Programmed death receptor 1 (PD-1), an inhibitory immune checkpoint receptor constitutively expressed on activated T cells, protects healthy cells from excessive inflammatory or autoimmune responses in combination with its ligand, programmed death ligand 1 (PD-L1). However, tumors can evade the antitumor activity of the immune system and continue to grow by inhibiting proliferation of T cells, promoting their apoptosis and reducing their secretion of proinflammatory cytokines, effects that are a consequence of binding of PD-1 to PD-L1 on tumor cells [7–10].

Immunotherapy using immune checkpoint inhibitors (ICIs), such as antibodies against PD-1 or cytotoxic T-lymphocyte-associated antigen-4 (CTLA-4), has recently emerged as a potential treatment for various malignancies, and PD-1/PD-L1 inhibitors have been approved by the Food and Drug Administration (FDA) as the first- or second-line treatments for urothelial carcinoma [11–14]. However, many cases remain unresponsive to these agents. Thus, a broad understanding of the immune system in the tumor microenvironment in UTUC and identification of reliable biomarkers would improve the identification of patients who are likely to benefit from treatment. Recently, five new B7 family ligands—B7 homologs 3 to 7 (B7-H3 to -H7)—were identified. Expression of the co-inhibitory molecule B7-H4 in cancer cells may be associated with tumor progression, due to its inhibition of T cell proliferation and cytokine production in the tumor microenvironment [15]. B7-H4 is over-expressed on tumor cells in various types of cancers and is negatively correlated with survival prognosis, including in colorectal, esophageal, gastric, kidney, ovarian and pancreatic cancers [15]. B7-H4 is a crucial inhibitor in step 7 of the cancer-immunity cycle, in which activated effector T cells kill their target cancer cell [16]. Therefore, it is likely that B7-H4 plays a significant role in the “immune escape” theory of tumors. Furthermore, B7-H4 might be an attractive immunotherapeutic target due to its high expression by tumors in combination with its low or absent protein expression in normal tissues. In fact, preclinical investigation into B7-H4-specific chimeric antigen receptor T cells, antibody-mediated blockade of B7-H4 and anti-B7-H4 drug conjugates has shown antitumor efficacy in mouse models [17].

Thus, investigations regarding the relationship between expression of B7-H4 and clinicopathological characteristics in tumor tissues might highlight the role of B7-H4 in UTUC.

## Methods

### Patients

We retrospectively analyzed data from 133 patients (95 men and 38 women; median age, 73 years; range 42–87 years) who underwent nephroureterectomy at our institution from 2005 to 2018. Patients had a diagnosis of clinical stage of cTanyN0M0 UTUC (renal pelvis, *n* = 61; ureter, *n* = 72). Preoperative staging of carcinomas was performed by computed tomography (CT) or magnetic resonance imaging (MRI) or both. Among the 81 patients who underwent nephroureterectomy in 2011 or later, 43 patients with a preoperative diagnosis of stage cT3-4N0M0 were also treated by lymphadenectomy. For lymphadenectomy, the regions removed depended on the type of tumor, as follows: for ureteropelvic tumors, medial to the ureter; for right-sided renal pelvic tumors and higher ureteral tumors, from the vena cava or right side of the aorta; for left-sided renal pelvic tumors and higher ureteral tumors, from the aorta; and in lower ureter tumors, the common/external/internal iliac and obturator region [18].

After surgery, 46 patients showed recurrence of the disease: The first site of recurrence was the bladder in 16 patients and the lymph nodes or distant organs in the remaining 30 patients. The latter group of patients was treated by cisplatin-based systemic chemotherapy consisting of gemcitabine and cisplatin or methotrexate, vinblastine, doxorubicin and cisplatin. At the first chemotherapy session, 17 of these patients received full-dose cisplatin and 13 received 50%–70% of the full dose because they had impaired cardiac or kidney function or a lower performance status or were older. At subsequent chemotherapy sessions, either the cisplatin dose was decreased or the patients were switched to carboplatin, depending on their cardiac and kidney function and performance status. Chemotherapy was unsuccessful in seven patients, so they were treated with the immune checkpoint inhibitor pembrolizumab, an anti-PD-1 agent. Patients were followed up for a median of 29 months (range 3–118 months) and underwent CT or MRI scans or both at 2- to 4-month intervals to check for metastasis. For this study, we reviewed the medical records until March 2021.

This study was conducted in accordance with the Declaration of Helsinki and was approved by the ethical review board of Dokkyo Medical University Hospital. Each patient signed a consent form that was approved by our institutional Committee on Human Rights in Research.

## Immunohistochemistry

Surgically resected tumor tissue specimens were sectioned at a thickness of 4 μm, fixed in formalin and embedded in paraffin. Immunohistochemistry was performed using the rabbit anti-B7-H4 antibody (Abcam, ab209242, Cambridge, the UK), anti-PD-L1 (OptiView PD-L1 [SP142]; Ventana Medical Systems, Inc.), anti-forkhead box P3 (Foxp3) antibody (Abcam, ab191416, Cambridge, the UK), anti-CD8 antibody (Leica Biosystems Newcastle Ltd, PA0183, Newcastle, the UK) and the mouse anti-T cell intracellular antigen 1 (TIA-1) as a biomarker for activated cytotoxic CD8 T cells [19–21] (Abcam, ab2712, Cambridge, the UK). Immunohistochemical staining was performed using the automated BOND-III system (Leica Biosystems Newcastle Ltd), according to the manufacturer’s instructions. B7-H4, CD8 and TIA-1 were examined by immunohistochemistry in all 133 primary tumors. In addition, in the 30 primary tumors that recurred in the lymph nodes or distant organs, PD-L1 and Foxp3 were also stained to enable comparison of the expression levels of the five molecules and the effects of systemic chemotherapy on them.

Computer-assisted cytometrical analysis with WinROOF image processing software (Mitani Corp., Tokyo, Japan) was used to calculate the proportion of the whole area that was positive for B7-H4 (Supplementary Figure 1); for a detailed description of the method, see our earlier publication [22]. Patients were then divided into a group with high expression of B7-H4 (proportion of total area positive for B7-H4: > 30%) and one with low expression (proportion of total area positive for B7-H4: < 30%); this process is also described in detail in an earlier paper [23].

For the remaining four molecules, the amount of staining in 1000 cancer and 1000 neighboring noncancer cells was assessed in 5–10 microscopic fields on 5–7 slide sections. Expression of these molecules in tumor cells (TC) and infiltrating immune cells (IC) was assessed on a scale ranging from TC- or IC-0 to TC- or IC-3, as follows: TC-0/IC-0, less than 1% staining; TC-1/IC-1, 1% to 5% staining; TC-2/IC-2, 5% to 30% staining; and TC-3/IC-3, greater than 30% staining. In accordance with our earlier study [24], this classification was then used to divide tumors into a group with low expression, which included TC-0, IC-0, TC-1 and IC-1, i.e., cells with either negative or weakly positive immunohistochemical staining, and a group with high expression, which included TC-2, IC-2, TC-3 and IC-3, i.e., cells with strongly positive staining. As a final step, response rate and overall survival were assessed in the two expression groups.

## Statistical analysis

Associations in the expression of B7-H7, PD-L1, Foxp3, CD8 and TIA-1, and clinicopathological findings were analyzed by Pearson’s χ^2^ test for contingency tables. Overall survival curves were created using the Kaplan–Meier method, and differences between the curves were assessed with the log-rank test. We examined prognostic factors affecting survival using Cox regression analysis. Analyses were performed with commercially available software, and *p* < 0.05 was considered to indicate significance.

## Results

**Fig. 1:**
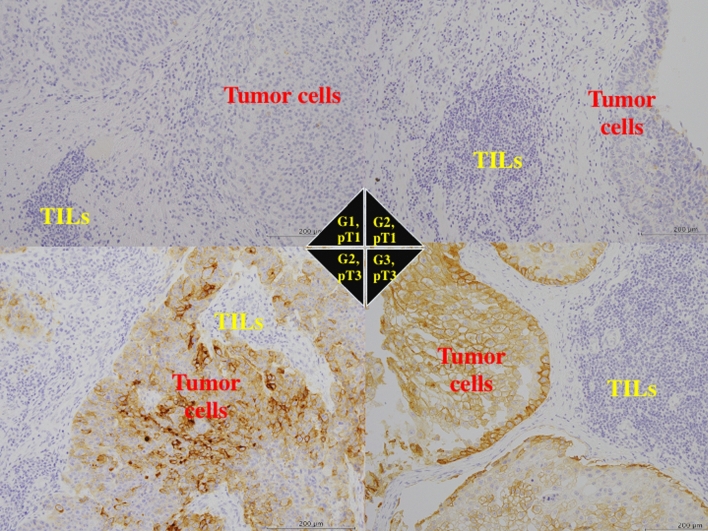
Immunohistochemistry of B7-H4. The tumor cells with well to mediate histological differentiation (G1/2) or lower stage (pT1/2) had very low expression for B7-H4. The tumor cells with poorer histological grade (G3) and higher stage (pT3) showed strong staining for anti-B7-H4 antibody. Tumor-infiltrating lymphocytes (TILs) showed no staining of B7-H4

**Fig. 2:**
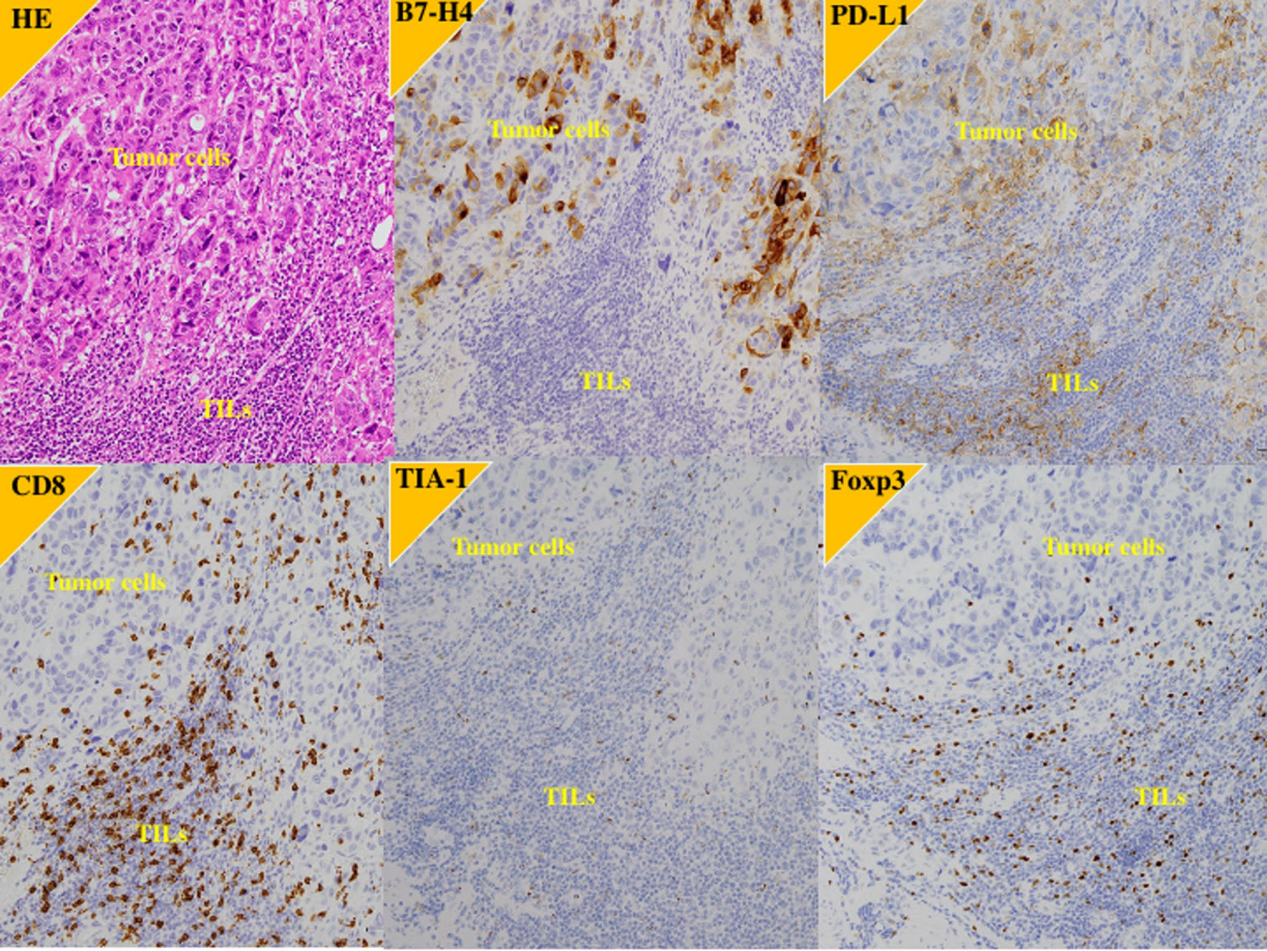
Immunohistochemistry of the primary tumor in a case of better response to pembrolizumab after failure of the first-line platinum-based chemotherapy. 61 y.o. male with right ureter urothelial carcinoma with grade 3, pT3N0M0. After right nephroureterectomy, the carcinoma recurred in retroperitoneal lymph node. B7-H4; high expression, PD-L1; high expression, CD8: high expression, TIA-1; high expression, Foxp3; high expression

**Fig. 3:**
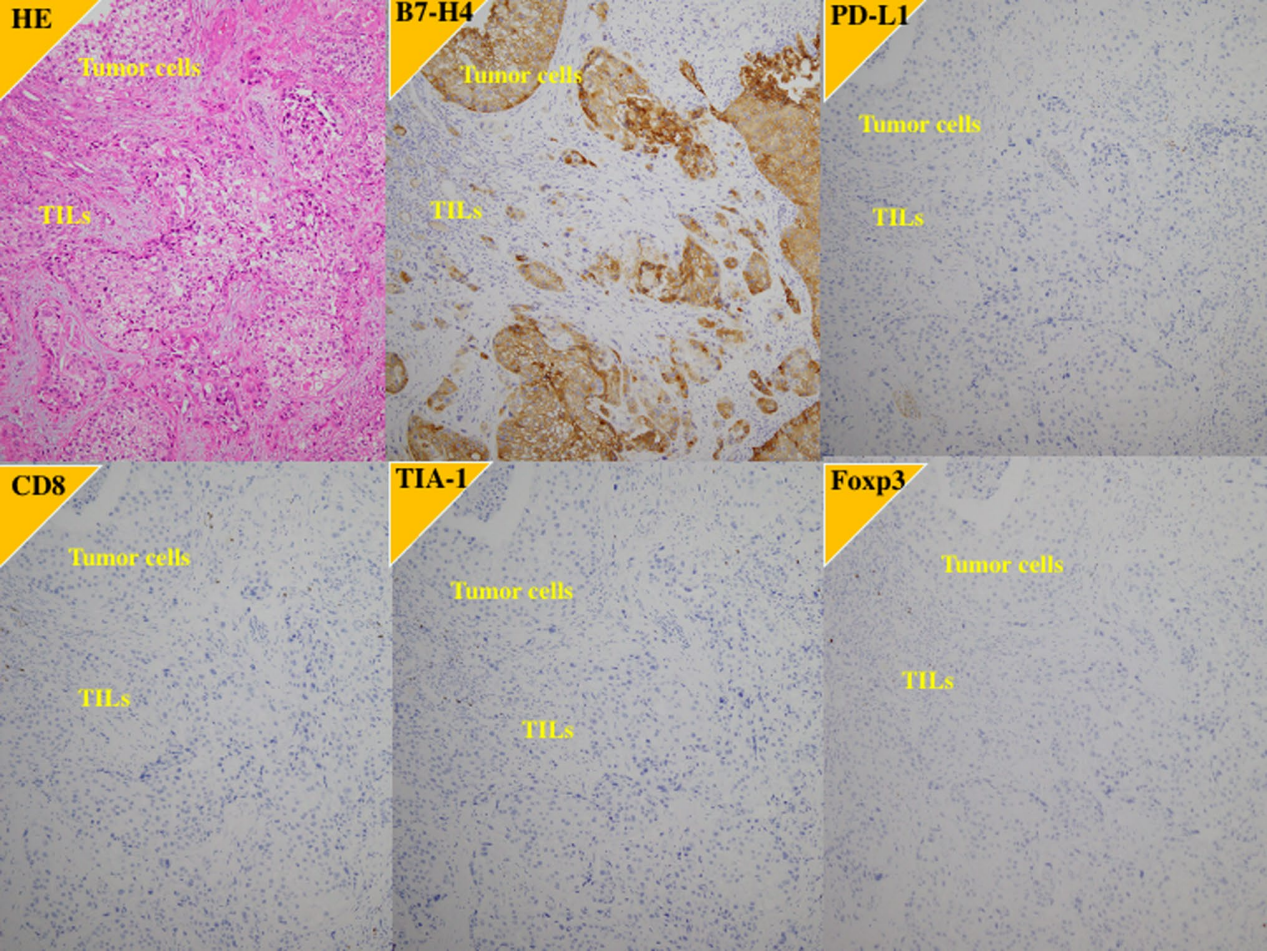
Immunohistochemistry of the primary tumor in a case of poorer response to the first-line platinum-based chemotherapy and the second-line pembrolizumab. 73 y.o. female with left ureter urothelial carcinoma with grade 3, pT3N0M0. After left nephroureterectomy, the carcinoma recurred in retroperitoneal lymph node. B7-H4; high expression, PD-L1; low expression, CD8: low expression, TIA-1; low expression, Foxp3; low expression

B7-H4 was mainly expressed on the membrane and in the cytoplasm of tumor cells, while normal urothelium and tumor-infiltrating lymphocytes showed no or very weak reactivity for anti-B7-H4 antibody (Fig. 1). When stained, PD-L1, Foxp3, CD8, TIA-1 were expressed diversely in tumor cells and tumor-infiltrating lymphocytes (Figs. 2, 3).

Expression of B7-H4 in tumors was related to histological grade (*p* < 0.0001), local invasion (*p* = 0.0005), regional lymph node involvement (*p* = 0.0011) and lymphovascular invasion (LVI) (*p* = 0.0001, Table 1).

**Table 1:** Relationship between pathological factors and expressions of B7-H4, CD8 and TIA-1

		Grade		pT		pN		LVI		B7-H4		CD8	
		1,2 (*n* = 75)	3 (*n* = 58)	< 1 (*n* = 33)	2–4 (*n* = 100)	0 (*n* = 115)	1,2 (*n* = 18)	0 (*n* = 62)	1 (*n* = 71)	Low (*n* = 70)	High (*n* = 63)	Low (*n* = 103)	High (*n* = 30)
B7-H4	Low (*n* = 70)	53	17	25	45	69	1	43	27				
	High (*n* = 63)	22	41	8	55	46	17	19	44				
	*p* value	< 0.0001		0.0005		0.0011		0.0001					
CD8	Low (*n* = 103)	58	45	22	81	91	12	48	55	60	43		
	High (*n* = 30)	17	13	11	19	24	6	14	16	10	20		
	*p* value	0.9253		0.4481		0.5687		0.9951		0.0244			
TIA-1	low (*n* = 110)	64	46	25	85	94	16	47	63	61	49	101	9
	High (*n* = 23)	11	12	8	15	21	2	15	8	9	14	2	21
	*p* value	0.6796		0.4855		0.4962		0.0290		0.0862		< 0.0001	

CD8 expression was not associated with histological grade, local invasion, regional lymph node involvement or LVI (Table 1). In contrast, TIA-1 expression correlated with LVI (*p* = 0.0290), but not with histological grade, local invasion or regional lymph node involvement (Table 1).

B7-H4 expression was positively correlated with CD8 expression (*p* = 0.0244), and weakly related to TIA-1 expression (*p* = 0.0862, Table 1). There was also a significant positive relationship between CD8 and TIA-1 (*p* < 0.0001, Table 1).

**Fig. 4:**
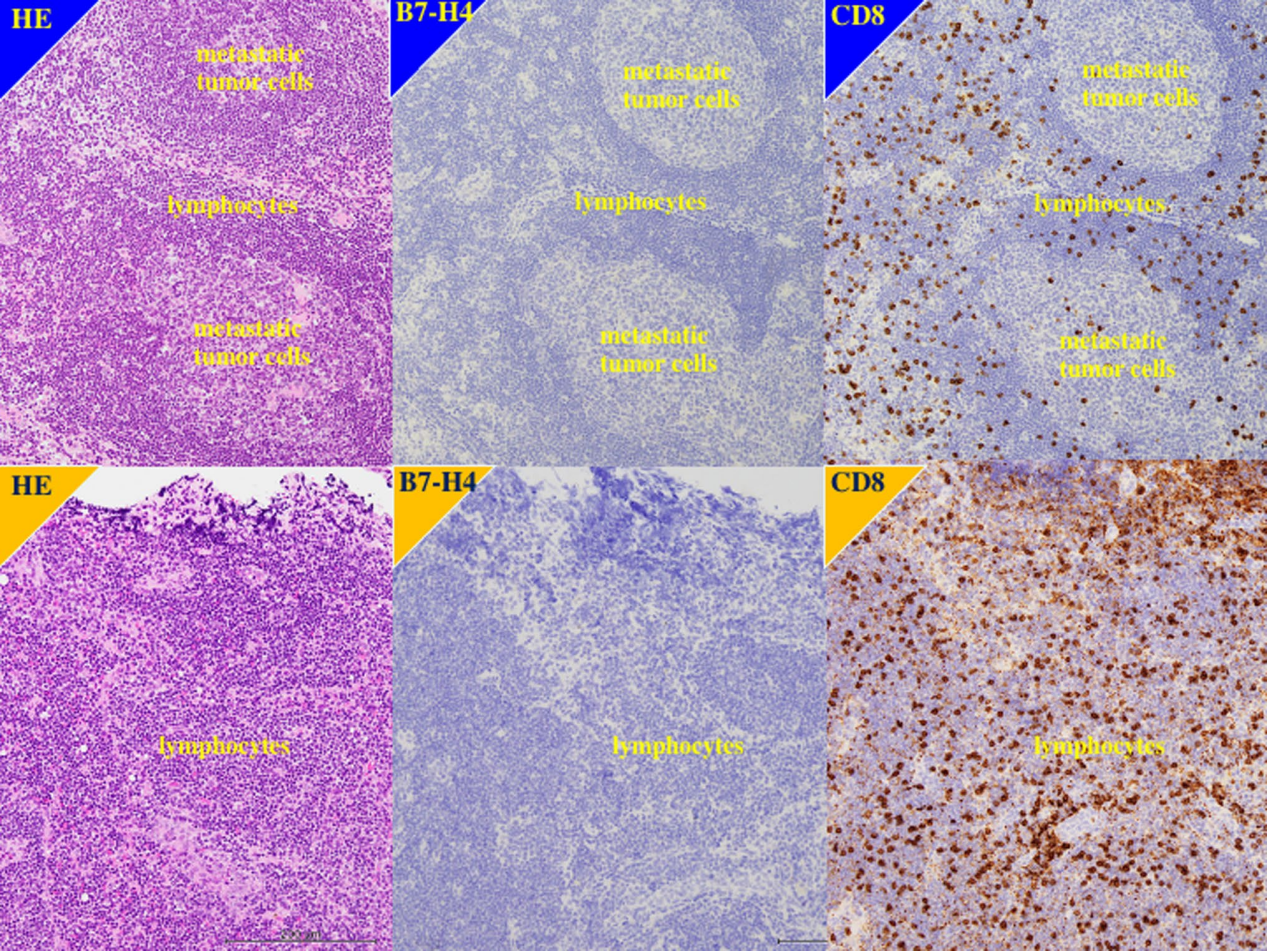
Immunohistochemistry in lymph node. Upper panels showed metastatic lymph node of the patients left renal pelvis urothelial carcinoma, 71 y.o. male, with grade 3, pT4N1M0. Metastatic tumor cells revealed no immunostaining for B7-H4, while lymphocytes showed positive immunostaining for CD8. Lower panels showed non-metastatic lymph node of right renal pelvis urothelial carcinoma, 87 y.o. female, with grade 3, pT3N0M0. Lymphocytes showed intense immunostaining for CD8, but negative immunostaining for B7-H4

In 18 patients, enlarged, 0.8- to 1.0-cm diameter lymph nodes were found at nephroureterectomy and diagnosed as metastases. In immunohistochemical staining, none of the metastatic lymph nodes and the non-metastatic lymph nodes was positive for B7-H4. In contrast, lymphocytes in metastatic and non-metastatic lymph nodes were positive for CD8 (Fig. 4).

Forty-six patients had tumors that recurred after nephroureterectomy; 16 tumors recurred in the bladder, and 30 in the retroperitoneal lymph nodes (RPLN), pelvic lymph nodes (PLN) or distant organs. Tumors with high B7-H4 expression were associated with postsurgical cancer recurrence in RPLN, PLN or distant organs, while those with low B7-H4 expression were associated with no recurrence or with recurrence in the bladder (*p* < 0.0001, Table 2). CD8 and TIA-1 expression was not associated with the site of first recurrence (Table 2).

**Table 2:** First recurrence site in 115 patients wihout regional lynph node metastastasis, and response for systemic therapy for metastatic lesions

			No recurrence (*n* = 69)		Bladder (*n* = 16)	RPLN/PLN* (*n* = 24)		Ditant organs (*n* = 6)
B7-H4	Low (*n* = 69)		54		11	3		1
	High (*n* = 46)		15		5	21		5
	*p* value		< 0.0001					
CD8	Low (*n* = 89)		55		13	18		3
	High (*n* = 26)		14		3	6		3
	*p* value		0.8994					
TIA-1	Low (*n* = 95)		54		14	21		6
	High (*n* = 20)		15		2	3		0
	*p* value		0.7839					

				PR*/SD* (*n* = 12)			PD* (*n* = 18)	
B7-H4		Low (*n* = 5)		5			0	
		High (*n* = 25)		7			18	
		*p* value		0.0056				
PD-L1		Low (*n* = 18)		5			13	
		High (*n* = 12)		7			5	
		*p* value		0.1362				
Foxp3		Low (*n* = 23)		9			14	
		High (*n* = 7)		3			4	
		*p* value		0.8605				
CD8		Low (*n* = 23)		7			16	
		High (*n* = 7)		5			2	
		*p* value		0.0837				
TIA-1		Low (*n* = 25)		8			17	
		High (*n* = 5)		4			1	
		*p* value		0.1282				
B7-H4 / PD-L1		Low / high (*n* = 3)		3			0	
		Low / low (*n* = 2)		2			0	
		High / high (*n* = 9)		4			5	
		High / low (*n* = 16)		3			13	
		*p* value		0.0142				
B7-H4 / Foxp3		Low / high (*n* = 1)		1			0	
		Low / low (*n* = 4)		4			0	
		High / high (*n* = 6)		7			4	
		High / low (*n* = 15)		3			12	
		*p* value		0.1085				
B7-H4 / CD8		Low / high (*n* = 2)		2			0	
		Low / low (*n* = 3)		3			0	
		High / high (*n* = 5)		3			2	
		High / low (*n* = 20)		4			16	
		*p* value		0.0023				
B7-H4 / TIA-1		Low / high (*n* = 2)		2			0	
		Low / low (*n* = 3)		3			0	
		High / high (*n* = 6)		2			4	
		High / low (*n* = 19)		5			14	
		*p* value		0.0111				

RPLN/PLN* : retroperitoneal lymph node/pelvic lymph node, PR*: partial response, SD*: stable disease, PD*: progressive disease

In the 30 recurrent tumors in lymph nodes or distant organs, analyses of expression by immunohistochemical staining revealed a weakly positive correlation of PD-L1 with Foxp3 (*p* = 0.0837) but not with B7-H4 (*p* = 0.3644), CD8 (*p* = 0.3915) or TIA-1 (*p* = 0.3644). Foxp3 was positively correlated with CD8 (*p* = 0.0331) but not with B7-H4 (*p* = 0.8447) or TIA-1 (*p* = 0.5650). Patients with primary tumors that showed a higher expression of B7-H4 and lower expression of CD8 responded worse to treatment than these with a lower expression of B7-H4 and higher expression of CD8 (*p* = 0.0056 for B7-H4 and *p* = 0.0837 for CD8; Table 2). When the combination of B7-H7 with each of the four molecules PD-L1, Foxp3, CD8 and TIA-1 was analyzed, patients with metastatic lesions where the primary tumors showed lower expression of B7-H4 and higher expression of PD-L1, CD8 or TIA-1 were found to have better treatment response (*p* = 0.0142 for PD-L1, *p* = 0.0023 for CD8 and *p* = 0.0111 for TIA-1; Table 2).

The seven patients who received pembrolizumab all had higher expression of B7-H4 in the primary tumors. In three of these patients, expression of PD-L1, CD8 and TIA-1 was higher, and the metastatic lesions showed partial response. In the remaining four patients, expression of PD-L1, CD8 and TIA-1 was lower, and the metastatic lesions showed worse treatment response; consequently, the survival time was shorter in these patients. We found no correlation between Foxp3 expression and response to treatment (Table 2).

**Fig. 5:**
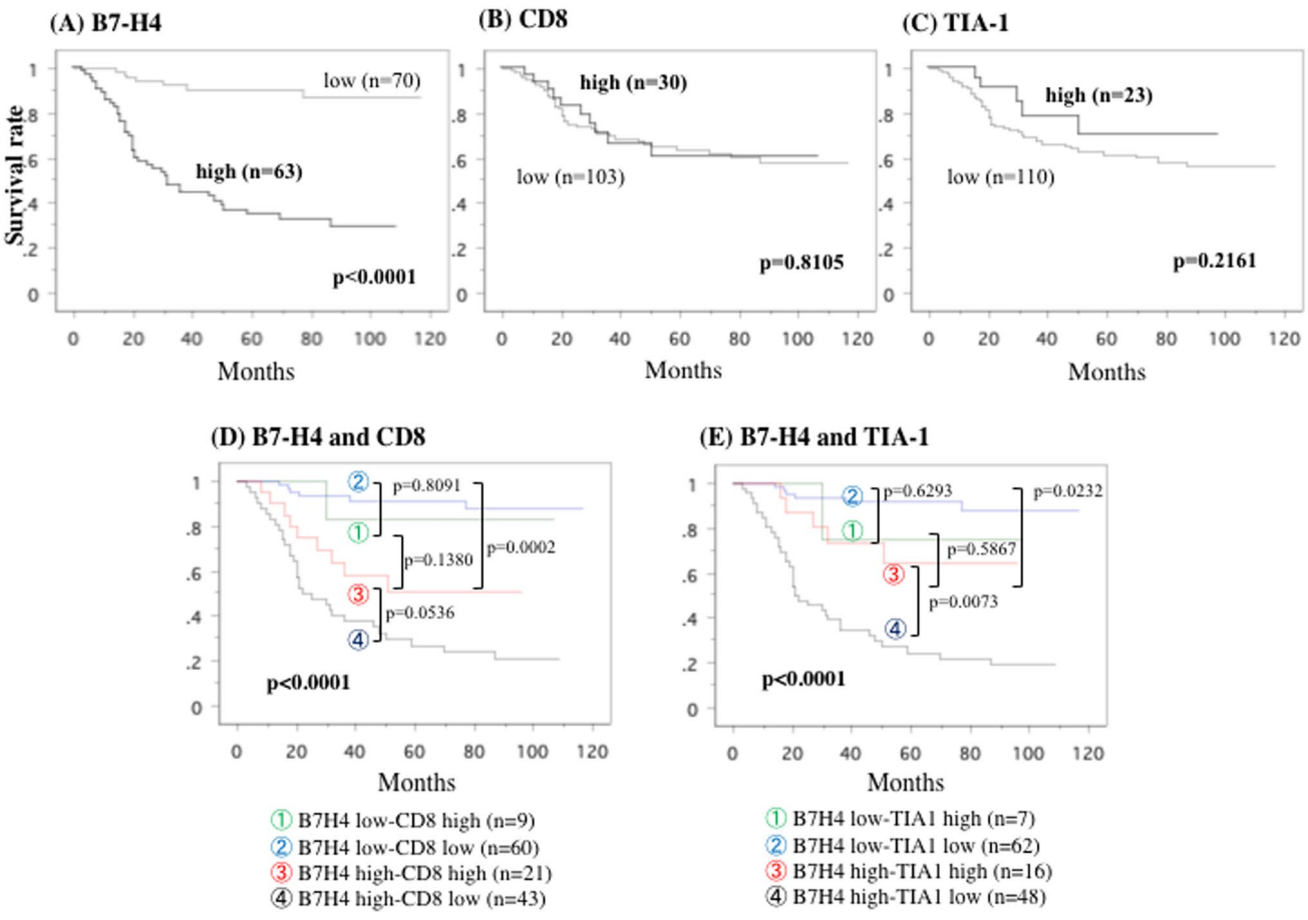
Overall survival curve. This overall survival curve is based on the expression status of B7-H4 (**a**), CD8 (**b**), TIA-1 (**c**), combination of B7-H4 and CD8 (**d**) and combination of B7-H4 and TIA-1 (**e**) in all patients. In Figure **d**, in comparison to the patients in type IV (B7-H4 high and CD8 low), the relative risk (RR) of the patients in type II (B7-H4 low and CD8 low) was 0.083 (95% confidential intervals (CI); 0.035–0.200, *P* < .0001), RR in type I (B7-H4 low and CD8 high) was 0.106 (95% CI; 0.014–0.776, *P* = .0271), and RR in type III (B7-H4 high and CD8 high) was 0.482 (95% CI 0.230–1.011, *p* = .0536). In Figure E, compared to the patients in type IV (B7-H4 high and TIA-1 low), the RR of the patients in type II (B7-H4 low and TIA-1 low) was 0.075 (95% confidential intervals (CI) 0.032–0.180, *P* < .0001), RR in type I (B7-H4 low and TIA-1 high) was 0.133 (95% CI 0.018–0.971, *P* = .0466), and RR in type III (B7-H4 high and TIA-1 high) was 0.277 (95% CI 0.108–0.707,* p* = 0.0073)

Kaplan–Meier estimates showed that higher B7-H4 expression in tumors was significantly related to shorter overall survival (*p* < 0.0001, Fig. 5a), but CD8 and TIA-1 expression was not related to survival (*p* = 0.8105 and *p* = 0.2161, respectively, Fig. 5b, c). Lower B7-H4 expression had no influence on survival when we divided the expression of CD8 or TIA-1 into two groups: low B7-H4/high CD8 or TIA-1 and low B7-H4/low CD8 or TIA-1 (Fig. 5d, e). In contrast, when we divided the high B7-H4–expressing tumors into two groups, high B7-H4/high CD8 and high B7-H4/low CD8, patients with high B7-H4/high CD8-expressing tumors had longer overall survival than these with high B7-H4/low CD8-expressing tumors (*p* = 0.0536, Fig. 5d). Similarly, of the tumors highly expressing B7-H4, those with a higher expression of TIA-1 (high B7-H4/high TIA-1) were associated with better survival than these with a lower expression of TIA-1 (high B7-H4/low TIA-1) (*p* = 0.0073, Fig. 5e).

In Cox univariate analysis, higher histological grade, higher pT stage, regional lymph node metastasis, positive LVI and higher B7H4 were associated with shorter overall survival. By Cox multivariate analysis, B7-H4 and LVI were significantly associated with shorter overall survival (Table 3).

**Table 3:** Cox regression analysis for various potential prognostic factors in overall survival

Variable	Unfavorable/favorable characteristics	No. of patients	Univariate (U)			Multivariate (M)		
			Relative risk	95% confidential interval	P value	Relative risk	95% confidential interval	P value
Grade	01–02-2003	58 / 68 / 7	2.77	1.59–4.81	0.0003	0.91	0.47–1.72	0.7568
pT	4,3,2/1 >	100/33	2.68	1.86–3.86	< 0.0001	1.36	0.84–2.21	0.2111
pN	2,1/0	18/115	2.62	1.74–3.95	< 0.0001	1.25	0.81–2.02	0.2995
LVI	1/0	71/62	6.28	2.94–13.42	< 0.0001	2.89	1.04–6.91	0.0404
CD8	Low/high	104/29	1.09	0.46–1.84	0.8115	1.46	0.28–1.71	0.4161
TIA-1	Low/high	110/23	1.78	0.22–1.42	0.2246	1.65	0.18–1.99	0.4080
B7-H4	High/low	63/70	9.48	4.25–21.16	< 0.0001	6.74	2.79–16.26	< 0.0001

## Discussion

The present study examined the expression levels of B7-H4, CD8 and TIA-1 in 133 patients with UTUC who underwent nephroureterectomy. B7-H4 was mainly expressed on tumor cells, whereas there was a diversity in the expression pattern of CD8 and TIA-1 in individual cases. We found the following: (1) higher expression of B7-H4 in tumor cells was associated with poorer histological differentiation, local invasion, poorer response to systemic chemotherapy and an independent prognostic factor for shorter survival; (2) in tumors with higher B7-H4 expression, higher CD8/TIA-1 expression was associated with better survival than lower CD8/TIA-1 expression. These observations suggest that elevated B7-H4 expression is related to cancer progression.

In the cancer-immunity cycle, B7-H4 plays an inhibitory role in step 7, the killing of cancer cells [16]. B7-H4 is the most critical mechanism of tumor evasion, inhibiting T cell proliferation, inducing T cell exhaustion and enhancing the activity of regulatory T cells [15–17]. We retrospectively analyzed data on patients with urothelial carcinoma of the renal pelvis or ureter with the aim to improve understanding of how B7-H4 is involved in regulating T lymphocytes. We found that expression of B7-H4 in tumor cells correlated positively with that of CD8 and TIA-1 and that in some tumors, the proportion of B7-H4-positive cells was correlated with the number of CD8-positive lymphocytes; this was particularly true in those cells with greater expression of PD-L1. Thus, we hypothesize that CD8 + T cells increase expression of B7-H4. Furthermore, among the patients with higher B7-H4 expression in primary tumors, those with higher CD8 or TIA-1 expression had a better response to systemic chemotherapy and/or second-line immunotherapy, and longer survival than these with lower CD8 or TIA-1 expression. These observations suggest an important role of CD8 T cells in controlling cancer and a clinicopathological link between expressions of B7-H4 and CD8/TIA-1 in this disease. CD8 T cells are one of the primary tumor-infiltrating immune cells that produce IFN-γ; they thereby contribute to an inflammatory environment that favors antitumor immunity and modifies the clinical response in cancer immunotherapies [25, 26]. CD8 T cell dysfunction is relatively common in solid tumors; therefore, several approaches have been suggested to increase CD8 T cell infiltration and restore the functions of CD8 T cells for cancer therapy [25, 26]. A previous study showed that CD8 expression increased during ICI treatment in patients with better treatment response [27]. The prognosis of bladder UC is better when high levels of CD8 T cells infiltrate the tumor site [28, 29]. In the present study, we found a positive relationship between B7-H4 and CD8/TIA-1 expressions. Currently, the molecular mechanism of effect of CD8 is uncertain; however, CD8 might infiltrate responsively in more aggressive tumors with poorer differentiation and a more invasive phenotype with higher B7-H4 expression. Thus, identifying the molecular mechanism of crosstalk between B7-H4 and cytotoxic CD8 T cells might open the pathway to exploring immunotherapies targeting B7-H4 in UTUC.

On the other hands, CD8 T cells may become inhibited or exhausted because long-term stimulation by antigens or metabolic reprogramming causes them to overexpress inhibitory receptors, including PD-1, leading to a decrease in their function and ability to multiply [30–32]. Exhausted CD8 T cells have decreased effector function and proliferative capacity and caused in part by overexpression of inhibitory receptors such as PD-1 [30–32]. Blockade of PD-1 reinvigorates T cell responses. Therefore, elucidating how T cell exhaustion contributes to the tumor microenvironment may help us understand the immunosuppressive mechanisms of tumors and develop immunotherapeutic interventions. In general, CD8-positive cell counts include both activated and exhausted CD8-T cells. TIA-1, a 17-kDa cytoplasmic granule-associated protein that is also designated GMP-17 (17-kDa granule membrane protein), might be involved in the signal cascade of Fas(CD95)-mediated apoptosis [19]. The GMP-17/TIA-1 molecule is expressed in lymphocytes possessing cytolytic potential, and anti-TIA-1 antibodies are thought to detect cytotoxic effector cells in lymphocyte-infiltrated tissue [20, 21]. Therefore, TIA-1 staining is considered a functional marker for activated cytotoxic CD8 T cells, with exhausted CD8 T cells being TIA-1 negative. As shown in Fig. 3, among the tumors with higher staining for CD8, some tumors showed lower staining for TIA-1, a marker of activated CD8 T cells, indicating that exhausted CD8 T cells were included within CD8 positive T cells. Impressively, in the present study, when the tumors highly expressing B7-H4 were divided into higher and lower TIA-1-expressing tumors, the primary tumors with higher TIA-1 expression were associated with longer survival rates and better responses of metastatic lesions to systemic chemotherapy than these with lower TIA-1 expression. These findings suggest that activated cytotoxic CD8 T cells play a role in suppressing the cancer. Measuring B7-H4 and TIA-1 expression might enable the prediction of outcomes of patients with UTUC.

Regarding the site of initial postoperative recurrence in patients with pTany pN0-M0 tumors at the time of nephroureterectomy, higher expression of B7-H4, but not CD8 or TIA-1, was related to postoperative recurrence in lymph nodes or distant organs. In patients in whom tumors first recurred in the bladder, many such tumors were detected as early superficial tumors and could be treated by transurethral resection. In contrast, the prognosis of the patients in whom the tumors recurred in lymph nodes and/or distant organs was shorter than we expected, probably because many such metastatic lesions were resistant to systemic therapy. In this study, among the 30 tumors that recurred in lymph nodes and/or distant organs, those in patients in whom the primary tumor had higher B7-H4 and lower CD8 expression showed a poorer response to first-line platinum-based chemotherapy than these with lower B7-H4 and higher CD8 expression. Furthermore, in the seven patients who received pembrolizumab for the tumors after failure of prior first-line platinum-based chemotherapy, three patients whose primary tumors had higher B7-H4, higher PD-L1 and higher CD8/TIA-1 expression showed partial responses to metastatic lesions, while the four whose tumors had higher B7-H4, lower PD-L1 and lower CD8/TIA-1 expression showed poorer responses. In addition, higher B7-H4 expression was a significant determinant for shorter overall survival according to the multivariate analysis. Cytotoxic CD8 T cells can target and kill cancer cells, and immunotherapies are being used clinically to boost this process at the tumor site and maintain an effective antitumor response [25, 26]. Our findings suggest that suppression of B7-H4 and recruitment and accumulation of activated cytotoxic CD8 T cells in the tumor microenvironment might lead to enhanced cytotoxic T-lymphocyte-mediated killing of infected cells. In an animal model of bladder UC, an antibody blocking B7-H4 was recently reported to enhance IFN-γ secretion by CD4 T cells and CD8 T cells, decrease tumor size, increase CD8 T cell infiltration in the bladder and decrease tumor-infiltrating regulatory T cells (Tregs), indicating that anti-B7-H4 treatment increased CD8 T cell activation as a mechanism of immune activation [33]. Thus, further mechanistic investigation of B7-H4 in UTUC is necessary to elucidate whether targeting B7-H4 genetically results in increased proliferation of CD8 T cells and decreased levels of exhaustion transcription factors.

Understanding differences in expression between primary tumors and metastatic lymph nodes lesion is important because the latter is targeted by chemotherapy and immunotherapy. In our sample, 18 patients were found to have metastatic lymph nodes at nephroureterectomy. It has been suggested that patients scheduled to undergo nephroureterectomy for later stage UTUC should be treated with neoadjuvant chemotherapy but that preoperative chemotherapy may not be unnecessary in earlier stage UTUC because the risk of lymph node metastasis is low [34, 35]. Tumor staging is inaccurate preoperatively [34, 35]. In our study, the metastatic lymph nodes were detected during surgery in all 18 patients, and none was identified by preoperative imaging. Although we hypothesized that the metastatic tumor cells in lymph node might had positive immunostaining for B7-H4, our immunohistochemical findings showed that metastatic tumor cells in smaller lymph nodes were not positive for B7-H4, but lymphocytes had higher staining for CD8. Thus, we suggest that either B7-H4 is involved in migration of cells from the primary tumor or its expression increases in metastatic lymph nodes at a later stage. To clarify this issue, future studies need to evaluate B7-H4 expression in larger metastatic lymph nodes and distant metastatic lesions that can be identified by preoperative imaging.

Numerous antigens, including self-antigens, are expressed by tumors, but infiltrating (CD4 + CD25 + Foxp3 +) Treg cells dampen the immune response, stopping it from attacking tumor cells [36]. B7-H4 is involved in this process by increasing Treg activity [37]. In addition, studies have shown that if macrophages in tumors express B7-H4, they reduce inflammation and T-cell proliferation in tumors and increase the production and function of Treg cells [15–17]. Treg cells are hypothesized to be regulated primarily by Foxp3 [38, 39]. In our study, we showed a weak correlation of expression of Foxp3 in metastatic lymph nodes with expression of PD-L1 but not with expression of B7-H4, CD8 or TIA-1 or with treatment response. Future studies are needed to determine CD4 + CD25 + Foxp3 + Treg cells in metastases.

Although the detailed mechanisms of B7-H4 expression in tumor cells remain unclear, tumor cells and T cells appear to interact in the regulation of B7-H4 expression, a process that is similar to the regulation of PD-L1 expression by innate and adaptive immune resistance mechanisms in the tumor microenvironment [8, 9]. As a result of these innate and adaptive immune resistance mechanisms, T cell receptor (TCR) recognition of the cognate antigen presented by major histocompatibility complex (MHC) molecules on the surface of cancer cells results in T cell activation. T cells then produce interferon (IFN)-γ and other cytokines [8, 9]. Cancer cells and other cells in the tumor microenvironment cause reactive expression of PD-L1, which turns off the T cells that are trying to attack the tumor, so they remain at the margin of the cancer [8, 9]. T cells are activated by cognate antigen presented by cancer cells. Cancer cells might sense that they are under attack from T cells by recognizing IFN-γ, which causes the reactive expression of B7-H4, which then turns off the antitumor T cell responses. On the other hands, multiple lines of evidence support an antitumor immunity by crosstalk of cytokines such as IFN-γ and PD-L1 in the tumor microenvironment, which allows cancers to resist the effects of endogenous tumor-specific T cells [40, 41]. Constitutive activation of oncogenic pathways such as the Akt/mammalian target of rapamycin (mTOR) pathway in tumor cells has also been reported to lead to increased glycolysis and elevated uptake of extracellular glucose. The resultant depletion of extracellular glucose causes dysfunction of tumor-infiltrating T cells and suppresses antitumor immunity, and PD-L1 expression by tumor cells promotes glycolysis through constitutive activation of the Akt/mTOR pathway [25, 26]. Thus, our findings indicate that increased B7-H4 expression might be linked to enhancement of tumor cell growth and anticancer immunity. To elucidate the molecular mechanism of B7-H4 activation in the tumor microenvironment, in the future, we need to measure cytokines such as IFN-γ, examine oncogenes/oncoproteins in pathways such as the Akt/mTOR pathway and perform functional studies with anti-B7-H4 antibody in cell lines or animal models.

The limitations of the present study were its relatively small patient cohort and short follow-up period, which prevent us from drawing definitive conclusions from the results. Since immunity is influenced by a complex set of tumor, host and environmental factors that govern the strength and timing of the anticancer response, a broader view of cancer immunity is required. The mechanisms underlying B7-H4-inducing enhancement of antitumor immunity and suppression of T cell immunity should also be studied in the future. Inflammatory cytokines within the tumor microenvironment, such as interleukin (IL)-2, IL-6, IL-10, IFNs and tumor necrosis factor α, have also been shown to induce B7-H4 expression on both tumor cells and monocytes/macrophages [15–17]. The shift toward immunotherapy targeting tumor cell-associated immune checkpoint molecules is likely to increase, so biomarkers predicting the efficacy of ICIs such as pembrolizumab may help identify patients with UTUC who are unlikely to benefit from these drugs, thus avoiding treatment failure. Our data highlight the importance of the interaction of B7-H4-related pathways with cytotoxic CD8 T cells in the tumor microenvironment. A larger cohort should be tested to establish the blockade of B7-H4 signaling as a novel treatment strategy for UTUC.

## Supplementary Information

Below is the link to the electronic supplementary material.

Supplementary Figure 1. Computer-assisted semiquantitative analysis for B7-H4 immunostaining. (A). Low immunostaining for B7-H4. (C). High immunostaining for B7-H4. The right panels show a low and a high WinRoof digital image, with green corresponding to the area of B7-H4-positive staining; low B7-H4 (B) and high B7-H4 (D) (TIFF 1142 KB)

## Abbreviations

B7-H4: B7 homolog 4
CD: Cluster of differentiation
CT: Computed tomography
CTLA-4: Cytotoxic T-lymphocyte-associated antigen-4
FDA: Food and Drug Administration
Foxp3: Forkhead box P3
GC: Gemcitabine and cisplatin
GMP-17: Granule membrane protein
ICI: Immune checkpoints inhibitor
IFN: Interferon
IL: Interleukin
LVI: Lymphovascular invasion
MDSC: Myeloid-derived suppressor cell
MHC: Major histocompatibility complex
MRI: Magnetic resonance imaging
mTOR: Mammalian target of rapamycin
MVAC: Methotrexate, vinblastine, doxorubicin and cisplatin
PD-1: Programmed cell death protein 1
PD-L1: Programmed cell death 1 ligand 1
PLN: Pelvic lymph nodes
pT: Pathological tumor
RPLN: Retroperitoneal lymph nodes
SUVmax: Pretreatment maximum standard glucose uptake
TAM: Tumor-associated macrophage
TCR: T cell receptor
TIA-1: T cell intracellular antigen 1
TIL: Tumor-infiltrating lymphocyte
TNF: Tumor necrosis factor
TNM: Tumor-lymph node metastasis classification of malignant tumors
Tregs: Regulatory T cells
TUR: Transurethral resection
UC: Urothelial carcinoma
UTUC: Upper tract urothelial carcinoma
