# Supplementary figures and images for: Elevated expression of B7 homolog 4 is associated with disease progression in upper urinary tract urothelial carcinoma
Source: Cancer Immunol Immunother. 2021 Jul 18;71(3):565–78. doi: 10.1007/s00262-021-03011-5 (PMC8854319; doi:10.1007/s00262-021-03011-5)

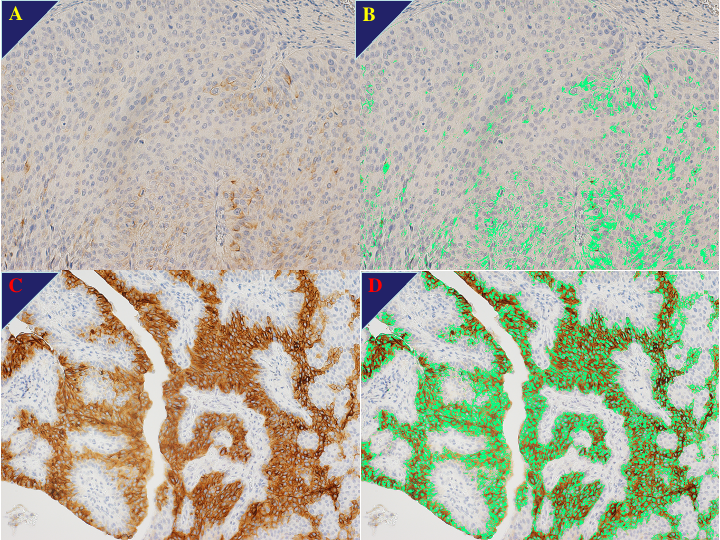

Supplement: Supplementary file 1 — Supplementary Figure 1. Computer-assisted semiquantitative analysis for B7-H4 immunostaining. (A). Low immunostaining for B7-H4. (C). High immunostaining for B7-H4. The right panels show a low and a high WinRoof digital image, with green corresponding to the area of B7-H4-positive staining; low B7-H4 (B) and high B7-H4 (D) (TIFF 1142 KB) [file 262_2021_3011_MOESM1_ESM.tiff]
